# Transfusion associated graft versus host disease in an immunocompetent individual following coronary artery bypass grafting

**DOI:** 10.4103/0972-5229.43681

**Published:** 2008

**Authors:** Girish T. Nagendra, Ramakrishna M. N., Devi Prasad Hegde, Sharad Damodar, Ratan Gupta

**Affiliations:** **From:** Department of Critical Care, Narayana Hrudayalaya Institute of Medical Sciences, 258/A, Bommasandra, Anekal Taluk, Bangalore, India

**Keywords:** Cardiopulmonary bypass, engraftment, immunocompetent, irradiation, transfusion

## Abstract

Transfusion associated graft versus host disease (TA-GVHD) is a rare but commonly fatal complication of transfusion of cellular blood products, which usually occurs in immunosuppressed individuals following transfusion and subsequent engraftment of viable T lymphocytes. Very rarely it may arise in apparently immunocompetent individuals. The clinical syndrome consists of fever, skin rash, diarrhoea, hepatic dysfunction, and bone marrow aplasia. The outcome is nearly always fatal. We present here a case report of fatal TA-GVHD in a “presumed” immunocompetent patient, post coronary artery bypass grafting surgery after transfusion of blood products. The patient died 24 days after transfusion.

There is a perceived increased risk of TA-GVHD following bypass grafting and other surgical procedures where cardiopulmonary bypass is required. TA-GVHD is probably underreported and the incidence is felt to be too low to warrant routine irradiation of cellular products for this group of patients. Clinicians, pathologists, and transfusion centers should be aware of this rare but devastating complication of blood transfusion after cardiac surgery.

## Introduction

Graft-versus-host disease (GVHD) is the clinical syndrome ascribed to the inflammatory reaction mounted by the donor cells against the host organs.[1] It was first described in humans after bone marrow transplantation (BMT) in 1959. Since then, it has been described in solid organ transplantation, blood transfusion and maternal-fetal transfer of leukocytes.

Transfusion-associated GVHD (TA-GVHD) is a dreadful, albeit infrequent complication of blood transfusion,[2] first reported in 1955. In spite of GVHD being a well-defined syndrome, the diagnosis of TA-GVHD is often delayed because of lack of awareness and the seemingly non-specific manifestations. The relative rarity of this syndrome prompted us to share our experience of managing a post-Coronary artery bypass patient at our unit.

## Case Report

A 60-year-old male was admitted to our hospital with congestive cardiac failure. After investigating him, he was diagnosed to have triple vessel disease with severe left ventricular dysfunction (LVEF- 35%) and moderate mitral regurgitation. His pre-operative laboratory tests showed: Hb - 14.7g/dl, TC – 6500 cell/cu.mm, Platelets – 222 thousands/cu.mm; Na – 140 mEq/L, K - 4.8 mEq/L, Urea – 9 mg/dl, Creatinine - 0.8 mg/dl, normal liver function tests; HbsAg, HIV, HCV - negative, VDRL - nonreactive.

He underwent elective coronary artery bypass grafting (CABG - on pump, 4 grafts). Surgery was relatively uncomplicated but in the immediate post-operative period the patient required high doses of inotropes and intra aortic balloon pump (IABP) augmentation to support him haemodynamically. He also lost 1600ml of blood through drains in the 1^st^ 24h for which he was re-explored, but was found to have generalized bleeding and no specific bleeders. After re-exploration he continued to bleed and the drain output was 1680ml over the next 72h before his drains were removed. During this period the blood products he received included 14 units of packed cells, 10 units of fresh frozen plasmas (FFPs), and 30 units of platelets, in an effort to control bleeding and to keep his haematocrit optimal.

He recovered slowly in the post-operative period and was transferred to the ward on the eighth post-operative day. At discharge to ward he had a Hb - 10.6g/dl, TC – 8400 cell/cu.mm, Platelets – 165 thousands/cu.mm; Na – 140 mEq/L, K - 4.5 mEq/L, Urea – 17 mg/dl, Creatinine - 0.9 mg/dl.

He had a non-progressing haematoma in the left groin, at the site of femoral arterial cannulation for IABP insertion.

Subsequently as the patient developed bilateral pedal edema with pain in the legs, anticoagulants were started suspecting DVT. He was re-admitted to the ITU on 21^st^ post-operative day with fever, leg pain and diarrhoea.

At readmission we found that he had a temperature of 102°C, pulse - 110/min, regular, and BP of 100/64 mmHg. He complained of leg pain and his venous doppler showed thrombus in the left common femoral vein and left proximal great saphenous vein. Investigations showed that he had a Hb - 10.3 g/dl, TC – 1500 cell/cu.mm, Platelets - 361thousands/cu.mm; Na – 133 mEq/L, K - 3.7 mEq/L, Urea – 19 mg/dl, Creatinine - 0.8 mg/dl, PT - 21.9, INR - 1.83, Total bilirubin - 2.2 mg/dl, Direct bilirubin - 1.4 mg/dl, SGOT – 149 U/L, SGPT – 98 U/L Alk Phos – 72 U/L.

He developed rashes three days after re-admission, which were initially present only in the peripheries, slowly spread all over his body and from being a macular rash transformed to blebs and erosions in the later stages. He was negative for all viral markers (Hepatic and retro viruses) and malarial, dengue and leptospira antigens. He grew enterococcus faecalis in blood which was covered with antibiotics according to the sensitivity pattern, but did not respond to treatment clinically.

**Figure 1:**
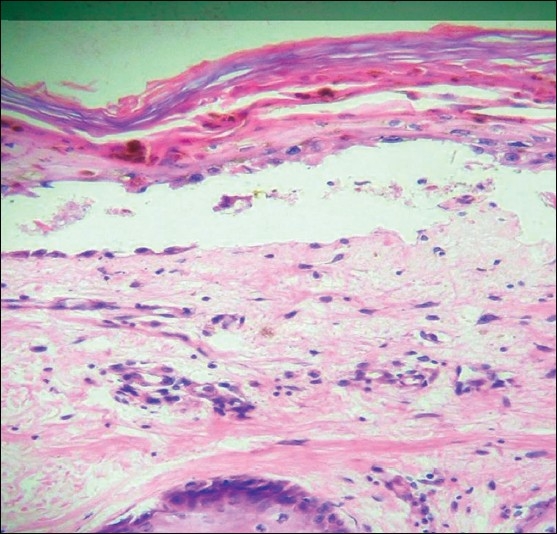
Mononuclear cell infiltration and inflammation of affected epithelium, with focal vacuolation of basal epithelial cells, (H&E, ×60)

His skin scrapings showed heavy candida and Tzank smear was negative. Skin biopsy showed mononuclear cell infiltration and inflammation of affected epithelium, with focal vacuolation of basal epithelial cells consistent with GVHD as shown in Figure 1. Bone marrow aspiration showed hypocellular marrow with presence of macrophages and increased iron stores.

**Figure 2:**
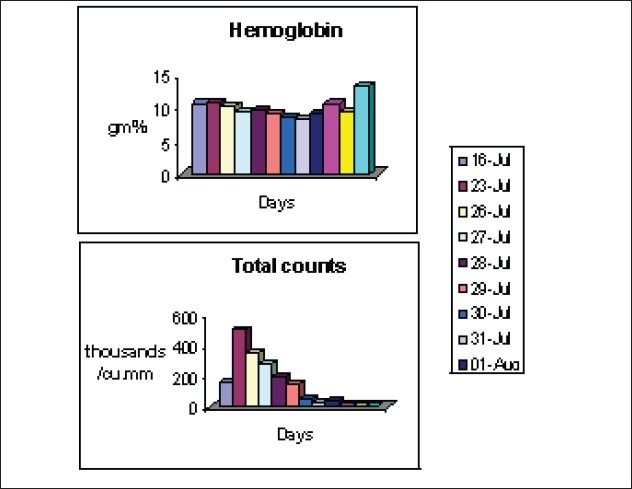
Change in hematologic parameters over day

He gradually dropped his total counts to 300 thousand/cu.mm [Figure 2] and became unstable haemodynamically requiring high doses of inotropes. After considering a diagnosis of TAGVHD, he was supported with steroids, G-CSF, ATG, antibiotics and antifungals with blood components. He had to be supported with RRT as he became anuric.

Despite full supportive care with blood products, broad spectrum antibiotics, mechanical ventilation, renal replacement therapy, he continued to have swinging pyrexia, worsening hepatitis, and bone marrow failure; he died 24 days after transfusion.

## Discussion

**Figure 3:**
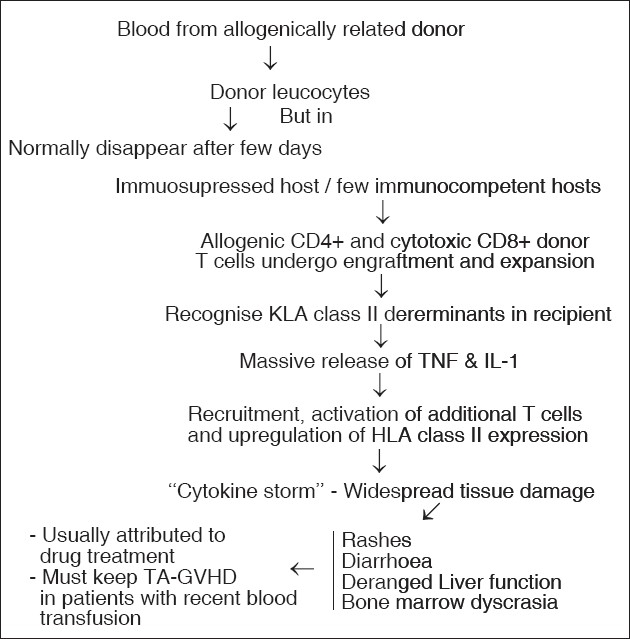
Pathogenesis of TA-GVHD

TA-GVHD arises 4 to 30 days after transfusion as a result of engraftment in the recipient of viable transfused lymphocytes from cellular blood products. It is typically seen in individuals with profound defects of cell-mediated immunity, but on rare occasions it is seen in apparently immunocompetent individuals[3] as explained in Figure 3. Death follows the development of TA-GVHD in more than 90% of cases[4] and there is no therapeutic agent of proved value. Irradiation of cellular blood products can electively prevent the development of this condition. Currently available blood filters are not deemed a suitable alternative.

TA-GVHD in immunocompetent individuals appears most commonly following CABG and other cardiovascular surgery in which cardiopulmonary bypass is required.[5,6] The reasons for this are not entirely clear but it is thought, that the use of relatively fresh blood with more viable lymphocytes increases the chance of engraftment.

Although it is clear that lymphocyte viability is affected by storage, the benefits of using fresh products after cardiopulmonary bypass are less well-documented. Some studies have shown a reduction in blood loss after cardiopulmonary bypass in recipients of fresh blood compared to stored blood, but there is a paucity of evidence for an overall clinical benefit or for reduction in homologous blood transfused.[7] In addition, cardiopulmonary bypass appears to produce a transient state of immunodeficiency defined by reduced mitogenic lymphocyte transformation and reduced interleukin 2 production. TA-GVHD in immunocompetent individuals occurs more commonly when a blood donor is homozygous for one of the recipient's major HLA types. It is suggested that because of the shared haplotype the host fails to recognize donor cells as foreign thus allowing engraftment. The viable T lymphocytes from the donor then mount a fulminant immune response against the host producing the clinical picture described above. Clearly, the risk of TA-GVHD in immunocompetent hosts increases in areas of low genetic diversity where the chance of shared HLA haplotypes with blood donors is increased.

Our patient, did not have any history of previous transfusion of blood or blood products. All the blood products transfused to him during the course of hospital were non-irradiated but none of them were obtained from his first degree relatives.

The reported frequency of one way matching or sharing HLA haplotype in a non-first degree relative in Japan ranges from 1 in 312[8] to 1 in 874 and as a result more than 200 cases of TA-GVHD have been reported in immunocompetent individuals in Japan. Greater HLA diversity probably accounts for the greatly reduced incidence of TA-GVHD in immunocompetent white patients. Guidelines on the indications for irradiation of cellular blood products to prevent the development of TA-GVHD, which have recently been published, do not recommend the routine irradiation of blood products for patients undergoing cardiopulmonary bypass. The use of cellular blood products during cardiac surgery has fallen over recent years owing to improved surgical techniques, preoperative haemodilution, the use of aprotinin, and red cell salvage techniques. In addition, the safety of blood transfusion has improved as a result of developments in compatibility testing and detection of viral infection. Despite these improvements, the potential for other rare but devastating complications of transfusion such as TA-GVHD persists.
